# Anticoagulant and Fibrinolytic Properties of Two Heparinoid Compounds Prepared from Shrimp Waste

**DOI:** 10.3390/foods12010066

**Published:** 2022-12-23

**Authors:** Jing Chen, Zhuo Wang, Xuejing Jia, Rui Li, Jianping Chen, Xiaofei Liu, Bingbing Song, Saiyi Zhong, Yi Qi

**Affiliations:** 1 College of Food Science and Technology, Guangdong Ocean University, Guangdong Provincial Key Laboratory of Aquatic Product Processing and Safety, Guangdong Province Engineering Laboratory for Marine Biological Products, Guangdong Provincial Engineering Technology Research Center of Seafood, Guangdong Provincial Science and Technology Innovation Center for Subtropical Fruit and Vegetable Processing, Zhanjiang 524088, China; 2 Shenzhen Research Institute, Guangdong Ocean University, Shenzhen 518108, China; 3 Collaborative Innovation Center of Seafood Deep Processing, Dalian Polytechnic University, Dalian 116034, China; 4 Guangdong Key Laboratory for Research and Development of Natural Drugs, The Marine Biomedical Research Institute, Guangdong Medical University, Zhanjiang 524023, China

**Keywords:** heparinoid, antithrombotic, anticoagulant, fibrinolytic, shrimp head

## Abstract

Heparinoid, a type of compound that has structures similar to heparin, has been found in marine organisms such as shrimp head. This shrimp waste products were used to prepare, characterize, and evaluate the antithrombotic effect of heparinoid. Two heparinoid compounds were obtained from shrimp head, and the main fraction F1 was →4)-GlcA-(1→3)-GalNAc-(1→ with Ara, while the minor fraction F2 composed mainly of the backbone as →4)-β-D-GlcA (or IdoA)-(1→4)-β-D-GlcN (or GlcNAc)-(1→. Both F1 and F2 could extend activated partial thromboplastin time and thrombin time concentration-dependently, and F2 has stronger activity than F1 at the same concentration. The potential anticoagulant mechanism of F1 and F2 may relate to their combination with more antithrombin III, which binds to and potentiates the action of antithrombin as well as inhibiting coagulation factors Xa and IIa, preventing blood clot formation. Furthermore, heparinoid F1 and F2 were found to have high fibrinolytic capability in vitro and in vivo via activating the self-fibrinolytic system. In conclusion, heparinoids (F1 and F2) derived from shrimp head wastes could be used as candidate compounds to prevent thrombosis while posing a lower hemorrhagic risk.

## 1. Introduction

Marine organisms are recognized as producers of natural products with special structure and various biological activities due to their unique environment. Marine shrimp has become a popular seafood with healthy protein and nutrients. *Penaeus vannamei* is one of the world’s three excellent shrimp aquaculture varieties; the output of *Penaeus vannamei* in China was about 1.2 million tons in 2020 [1] and it has a high economical value worldwide. A large amount of waste is produced, such as shrimp head, which accounts for 36% of the total shrimp weight during processing. However, shrimp heads are mostly wasted with limited utilization and low commercial value, even causing a waste of resources and environmental pollution. In particular, shrimp heads are rich in protein, lipids, chitin, carotenoids, pigments, enzymes, and sulfated glycosaminoglycans [2]. Cahú et al. [3] reported that bioactive substances were extracted from the head of *Litopenaeus vannamei* by the autolysis method, and 120 g protein hydrolysates, 25 g chitin, 195 mg carotenoid, and 79 mg glucosamine sulfate could be obtained from 1 kg of shrimp head, respectively. Thus, shrimp heads can be a good source of bioactive products with functional properties.

With the aging of the population and the change in people’s lifestyle and habits, thrombi have increasingly become a major global health problem. Thrombus formation is the primary cause of cardiovascular disease (CVD), and especially since the outbreak of the COVID-19 epidemic is more prone to thrombosis and thromboembolism events [4]. At present, there are three main types of antithrombotic therapy or intervention drugs commonly used in the market, including anticoagulant, thrombolytic and antiplatelet drugs. As a typical anticoagulant drug, heparin has an anticoagulant effect in vivo and in vitro, mainly through improving antithrombin, accelerating the inactivation of thrombin and Xa factor and preventing thrombosis. However, its side effects are obvious, and clinical symptoms such as bleeding and thrombocytopenia often occur [5]. Therefore, it is necessary to find a safe and effective compound as a substitute or supplement for heparin.

In recent years, some heparinoid compounds with heparin chemical structure and pharmacological properties have emerged, such as heparan sulfate, dermatan sulfate, chondroitin sulfate and some acidic ester polysaccharides, which are mainly used to prevent thrombosis. Heparinoid is a compound similar to heparin in structure and function, but its anticoagulant effect is mild and with various bioactivities. Saravanan et al. [6] reported that the activated partial thromboplastin time (APTT) of heparin-like compounds obtained from clams was 72 IU/mg, which may be an alternative source of heparin. Gomes et al. [7] reported that shellfish heparin compounds have a mild anticoagulant effect and the APTT assay showed that the anticoagulant activity was 36 IU/mg, which was five times lower than that of porcine heparin (180 IU/mg). Moreover, it can bind to antithrombin III and inhibit factor Xa and thrombin activity. Dietrich et al. [8] isolated natural low molecular weight heparin from *Litopenaeus vannamei*, and its anticoagulant effect is mainly through thrombin inhibition mediated by inhibitor Xa and heparin cofactor II. Palhares et al. [9] reported another chondroitin sulfate from *Litopenaeus vannamei* (sCS) with high anti-IIa activity and thrombin inhibitory effect mediated by heparin cofactor II. Consequently, it is possible to excavate heparinoids from shrimp head waste with good antithrombotic activity and low side effects.

In this study, heparinoid compounds were isolated from shrimp waste by enzymatic hydrolysis and anion exchange chromatography, then the structures of heparinoid compounds were characterized using methylation analysis combined with NMR. In addition, the anticoagulant, hemorrhagic and fibrinolytic effects of the two heparinoid compounds in vitro and in vivo, and their possible effects on thrombosis are also discussed. This provides insights into the potential development of heparinoid compounds to prevent thrombosis and also provides a good strategy for high-value utilization of shrimp waste.

## 2. Materials and Methods

### 2.1. Materials

Heparin standard was purchased from China National Institutes for Food and Drug Control (140817–201501) (Beijing, China). Heparinase I&II&III and chondroitinase ABC were purchased from Sigma-Aldrich (Merck KGaA, Darmstadt, Germany). Activated Partial Thrimboplastin Time Reagent (APTT), Prothrombin time Reagent (PT), Thrombin Time Reagent (TT) were purchased from Wuhan ZhongTai Biotechnology limited company (Wuhan, China). Anti-thrombin III (AT-III) was purchased from Chromogenic (West Chester, OH); Chromogenic Substrate for Factor Xa (S-2222, S-2765), and Chromogenic Substrate for Thrombin (S-2238) were purchased from Beijing Asnail Biotechnology (Beijing, China); Human Thrombin Enzyme was purchased from Hyphen-BioMed (95000 Neuville-sur-Oise, France). All other chemicals were of analytical grades or HPLC grades.

### 2.2. Animals

Beijing Hua Fu Kang Bio-Technology Company (SCXK 2019-0008) supplied SPF Kunming mice (5 weeks of age, 22 ± 2 g), mice were fed at 23 ± 2 °C under 12 h light/dark cycles with free water intake and feeding provided. All animal experiments complied with the Experimental Animal Ethics Committee of Guangdong Ocean University (SYXK2014-0053).

### 2.3. Extraction of Heparinoids and Purification from Shrimp Heads

**Figure 1:**
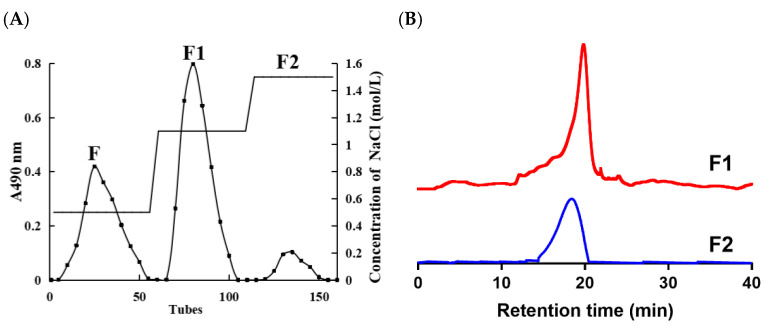
Elution curve of heparinoid in shrimp head of FPA98 Cl column (**A**), HPGPC chromatography of F1 and F2 (**B**).

Heparinoid compounds prepared from *Litopenaeus vannamei* shrimp heads were described in detail previously [10]. In brief, the shrimp heads were dried and crushed, and the powder was mixed with distilled water at a ratio of 1:10 (*m*/*v*), and then digested with 5% alkaline protease at 55 °C for 20 h. The enzymatic hydrolyses were cooled and centrifuged (8000 rpm/min for 20 min at 4 °C) after a boiling water bath for 10 min. The filtrate was taken and dynamically adsorbed by Amberlite FPA98 Cl anion exchange resin, and eluted with 0.5, 1.1, and 1.5 M NaCl solutions (Figure 1A). Then the elution components F, F1, and F2 were collected under a series of concentrations of NaCl (0.5, 1.1, and 1.5 M), respectively. Three fractions were precipitated with anhydrous ethanol (1 time the volume of the eluent) for further purification. The main precipitates were collected, concentrated, desalted, and freeze-dried to obtain purified heparinoids. Chemical analysis and molecular weight determination has been described in a previous study [10].

### 2.4. Disaccharide Composition Analysis

Disaccharide composition assay was carried out as previously described [11] with minor modifications. Briefly, the heparinoids F1 and F2 (20 μL, 20 mg/mL) were added with Ca(CH_3_COO)_2_ buffer (70 μL, pH 7.0), mixture heparinase I&II&III (100 μL, 0.4 IU/mL), and chondroitinase ABC (100 μL, 5 IU/mL). The reaction was placed for more than 48 h at 25 °C. Each of 120 μL of degradation solution was mixed with 20 μL NaBH_4_ buffer and performed over 4 h at 37 °C, these samples were filtered with 0.22 μm syringe filters. Then, the degraded samples were analyzed by strong anion exchange-high performance liquid chromatography (SAX-HPLC) on waters spherisorb SAX column (3 mm × 25 cm, 5 µm). Flow rate was 0.45 mL/min, with a column temperature of 50 °C and UV detection at 234 nm. The degraded samples separated using a gradient system, elution with mobile phase A (sodium phosphate 2 mM, pH 3.0) and B (sodium phosphate 2 mM, sodium perchlorate 1.0 M; pH 3.0), mobile phase B (0.5 min, 3–35% (20 min), 35–100% (50 min), 100%. The standard disaccharides include IS, IA, IIS, IIA, IIIS, IIIA, IVS and IVA were used for qualitative and quantitative assay.

### 2.5. Methylation Analysis

Heparinoids F1, and F2 were reduced by uronic acids, and methylated, hydrolyzed, and acetylated, then measured by GC-MS as in previous literature [12,13]. Briefly, samples (3 mg) were dissolved in DMSO (1.0 mL), then methylation reagent liquid A anhydrous alkaline solution and B methyl iodide solution was quickly added. After that the solution was reacted at 30 °C for 1 h reaction, and the reaction was terminated by adding 2 mL of ultrapure water. Then, these samples were hydrolyzed with 2 M trifluoroacetic acid (TFA) for 90 min and evaporated to dryness by a rotary evaporator. Residues were dissolved in 2 mL of ultrapure water, reacted with NaBH_4_ (60 mg), and neutralized with CH_3_COOH, added with C_4_H_6_O_3_ (1 mL) to be acetylated at 100 °C for 1 h. The acetylated samples were dissolved in CH_2_Cl_2_ (3 mL) and removed the upper aqueous solution. The partially methylated alditol acetates (PMAA) were examined by GC-MS (GCMS-QP 2010, Shimadzu, Japan). GC-MS condition: RXI-5 SIL MS column (30 m × 0.25 mm × 0.25 μm), temperature from 120 °C to 250 °C at a rate of 3 °C/min; 250 °C/min keep for 5 min; the inlet and detector temperature set at 250 °C. Helium gas was used as the carrier gas with a flow rate of 1 mL/min. Split ratio 5:1, injection volume 1 μL, the mass spectra were taken in SCAN mode at 300 eV from *m*/*z* 40–400. The MS progress started at 10 min and ended at 50 min.

### 2.6. NMR Analysis

F1 and F2 were individually dissolved in 0.5 mL D_2_O and repeated three times for hydrogen-deuterium exchange. Their ^1^H, ^13^C, Heteronuclear Single Quantum Coherence (HSQC), and Heteronuclear Multiple Bond Correlation (HMBC) spectra were recorded on a Bruker 600 MHz NMR spectrometer (Bruker BioSpin, Germany)at room temperature.

### 2.7. Anticoagulant Effects of Heparinoids

According to *Pharmacopoeia of the People’s Republic of China 2020* [14], the biochemical titer of anticoagulant activity was detected by chromogenic substrate method. Heparin standard was used as the positive group. APTT, PT, and TT assays were determined by Semiautomated Coagulation Analyzer (King Diagnostic, Guangzhou, China). In Brief, plasma (50 μL), samples of heparinoids (50 μL), and APTT reagents (50 μL) were added sequentially and incubated at 37 °C for 5  min before adding 25 mM CaCl_2_ (50 μL). The clotting time was taken as the value of APTT. Samples of heparinoids (50 μL) were mixed with plasma (50 μL) and incubated at 37 °C for 3 min, before adding 100 μL of pre-incubated PT reagent. The clotting time was recorded as the value of PT. Samples of heparinoids (100 μL) were mixed with 100 μL of plasma and incubated for 3 min at 37 °C, then 100 μL of reagent of TT was added and the clotting time was recorded as the value of TT. Chromogenic assays were carried out for samples of F1, F2, and heparin at 37 °C in a 96 well plate using Varioskan Flash (Thermo, Waltham, MA, USA). Then it was analyzed by the quantitative reaction parallel line method in the biological verification statistical program BS2000 to calculate the anti-Xa factor and anti-IIa factor titer of F1 and F2, with confidence limit (FL).

### 2.8. Hemorrhagic Effects of Heparinoids

The bleeding effects of F1 and F2 were evaluated by mouse cross-cut tail experiments [15]. Mice (22–24 g body weight) were divided into eight experimental groups randomly, including control group (saline), positive group (injected with heparin, 10 mg/kg of body weight), F1 group (2.5, 5, 10 mg/kg of body weight), and F2 group (2.5, 5, 10 mg/kg of body weight). After 30 min of circulation, the bleeding time was measured as follows: 3 mm tail tips of mice were amputated, the released blood was blotted onto filter paper every 30 s, and the time of bleeding cessation was noted.

### 2.9. Fibrinolytic Activity In Vitro of Heparinoids

Fibrinolytic activity was carried out by agarose fibrin plate assay as previously described [16,17], with slight adjustment. In a conical flask, agarose (15 mg/mL, 10 mL), fibrinogen (1.5 mg/mL, 5 mL) and thrombin (10 U/mL, 1 mL) were dissolved in PBS buffer (0.01 M pH 7.2). Then, the mixture medium was transferred to a Petri dish at room temperature and kept for 1 h. Next, 20 µL of sample with different concentrations (1 mg/mL, 5 mg/mL, and 10 mg/mL) were carefully injected into the wells (3 mm) in the fibrin plate. The plate was incubated for 18 h at 37 °C, stained for 2 min with mixture dye solution (0.25% Coomassie brilliant blue R-250, 10 mL acetic acid, and 50 mL methanol), and then decolorized in 4.5% methanol, 4.5% distilled water and 1% glacial acetate mixture, and the dissolved zones were measured with a Vernier caliper. The standard curve was prepared according to the dissolution area of urokinase with different concentrations (5, 10, 20, 40, 80, 160 and 200 U/mL), and the fibrinolytic activity of F1 and F2 was quantitatively calculated based on the dissolution zone area.

### 2.10. Effect of Heparinoids on Thrombosis and Fibrinolytic Activity In Vivo

According to the previous research [4,18], κ-carrageenan was used to induce mice tail thrombosis for estimating heparinoids antithrombotic activity. The animals were divided into 10 groups randomly, a control group (physiological saline), model group (physiological saline), positive group (injected with heparin, 2.5 mg/kg of body weight) and clopidogrel (10 mg/kg of body weight), F1 group (2.5, 5, 10 mg/kg of body weight), F2 group (2.5, 5, 10 mg/kg of body weight). Each group was administered by the intraperitoneal route of 0.1 mL/10 g of body weight injected volume. After 1 h, the blank control group was injected with the same dose of saline, and the other groups were injected with 0.2% carrageenan solution intraperitoneally (0.1 mL/10 g of body weight) and the mice were placed at 20 ± 2 °C environment temperature. The black tail formation of the mice was observed after 24 h and 48 h, and the lengths of the thrombus tails were measured by the vernier caliper. The whole thrombus tail was collected and fixed in 4% paraformaldehyde solution. Following dehydration, the tail tissue samples were paraffin embedded, sectioned, stained with haematoxylin-eosin (H&E) before being observed under a Leica DM3000 microscope (Wetzlar, Germany). Blood was collected from the eyeballs of the mice in each group, then tissue plasminogen activator (t-PA), plasminogen activator inhibitor-1 (PAI-1), and fibrinogen degradation product (FDP) levels in the blood were determined according to the ELISA kit (Shanghai Enzyme-linked Biotechnology, Shanghai, China).

### 2.11. Statistics Analysis

All data are presented as mean ± standard deviation (SD) of three technical replicates. The difference in statistical analysis was assessed by one-way ANOVA, Tukey’s multiple comparisons test and Dunn’s multiple comparisons tests.

## 3. Results

### 3.1. Preparation and Chemical Analysis of Two Heparinoids from Shrimp Heads

Through enzymatic hydrolysis, isolation, and purification of shrimp heads, three fractions named F, F1, and F2 were obtained (Figure 1A). The yield of F1 and F2 were 347 mg/kg and 91.67 mg/kg, respectively, which is appreciable considering the heparin extracted from other marine tissues. Dietrich et al. [8] reported that shrimp head can be used as a raw material for preparing low molecular weight heparin with a yield of 32 mg/kg. Relatively, glycosaminoglycan yield extracted from *Penaeus vannamei* processing waste was 79 mg/kg by Cahú et al. [3]. In addition, the glycosaminoglycan content of F1 and F2 was more than 80%. Meanwhile, the molecular weight (MW) of F1 and F2 was measured by GPC (gel permeation chromatography) as 7.8 kDa and 19.8 kDa (Figure 1B), respectively. The MW of F2 was similar to crab heparinoid (19 kDa) and lower than that of mammalian heparin (30 kDa) [19].

### 3.2. Disaccharide Composition of Two Heparinoids from Shrimp Head

**Figure 2:**
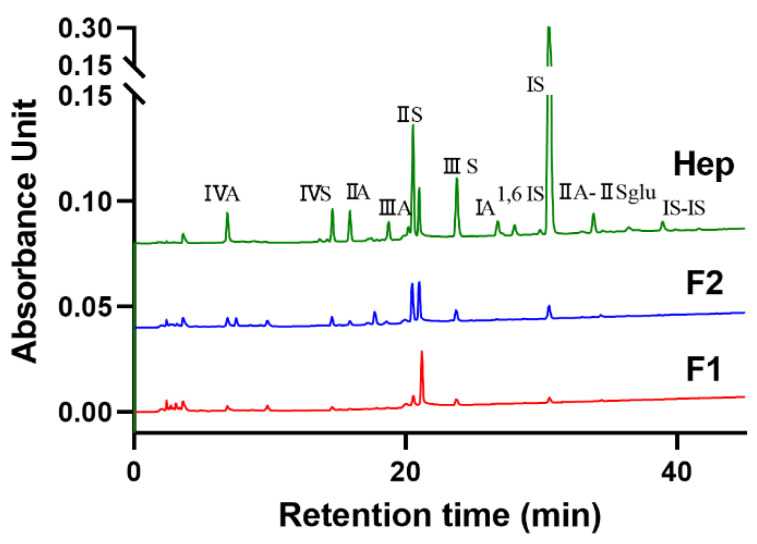
Disaccharides composition of heparinoid F1 and F2 by SAX-HPLC; IVA (ΔUA-GlcNAc), IVS (Δ UA-GlcNS), IIA (ΔUA-GlcNAc6S), IIIA (ΔUA2S-GlcNAc), IIS (ΔUA-GlcNS6S), IIIS (Δ UA2S-GlcNS), IA (ΔUA2S-GlcNAc6S), IS (ΔUA2S-GlcNS6S), IIA-IISglu (ΔUA-GlcNAc6S-GlcA-GlcNS3S6S).

To characterize the structure of heparinoid F1 and F2, two heparinoid compounds were treated with heparanase and chondroitinase, and disaccharides unit production were detected by SAX-HPLC (Figure 2). As shown in Table 1, there were different disaccharide compositions between F1 and F2,F1 mainly consisting of ΔUA2S-GlcNS, 6S (13.18%), ΔUA-GlcNS, 6S (26.56%), ΔUA2S-GlcNS (16.64%), ΔUA-GlcNS (7.97%) and ΔUA-GlcNAc (10.93%), while F2 was composed of the ΔUA2S-GlcNS, 6S (12.36%), ΔUA-GlcNS, 6S (28.97%), ΔUA2S-GlcNS (9.93%), ΔUA-GlcNS (6.39%), ΔUA2S-GlcNAc (11.17%) and ΔUA-GlcNAc (7.97%). Among them, the proportion of trisulfated disaccharide units for F1 and F2 was 13.18% and 12.36%, respectively, compared with 60.1% for porcine heparin, 89.4% for sheep and 96.2% for bovine heparin, and its lower trisulfated disaccharide units that may be classified as heparan sulfate. The content of disulfate disaccharide in F1 and F2 was relatively high with a proportion of 43.2% and 38.9%, respectively. F1 and F2 contained a small amount of monosulfate disaccharide, accounting for 11.94% and 21.05%, respectively. In addition, the non-sulfated disaccharide ratio of F1 was 10.93%, which was higher than 7.97% of F2, and the degree of sulfation was higher in F2. This finding is consistent with the disaccharide composition of mixed heparin/heparan sulfate in the head of *Litopenaeus vannamei* reported by Brito et al. [20], in which 73% of the GlcN units are N-sulfated and 6-sulfated, and only 22% are N-acetylated units.

### 3.3. Methylation Analysis

**Figure 3:**
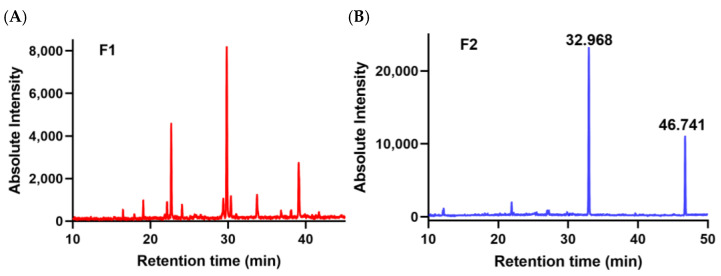
Total ion chromatogram of F1 (**A**) and F2 (**B**).

Detailed glycosidic characteristics are very necessary according to the close relationship between its linkages and bioactivity [21]. Acidic sugars of F1 and F2 were reduced to equivalent neutral sugars, and methylation products were further determined by GC-MS [12]. The glycosidic bonds of F1 and F2 were analyzed by methylation (Figure 3A,B). There was a difference in composition between F1 and F2 when comparing the CCRC (Complex Carbohydrate Research Center) Spectral Database of PMAA (Partially methylated alditol acetate) (uga.edu). F1 was mainly composed of a glucose formed by large sequences of →4)-Glc-(1→, residues of →3, 6)-Galp-(1→ and lower proportion Araf were also identified (Table 2). These residues are similar to the chain of chondroitin sulfate (CS), which underwent uronic acid reduction and methylation, and GC-MS analysis revealed that the main glycosidic linkage was →4)-Glc-(1→ [22]. F2 had two types of glycosidic bonds, including →3)-GlcNAc-(1→ and →4)-GlcA-(1→ residue composition (Table 2). Overall, most of these results need further confirmation based on NMR analysis.

### 3.4. NMR Analysis

**Figure 4:**
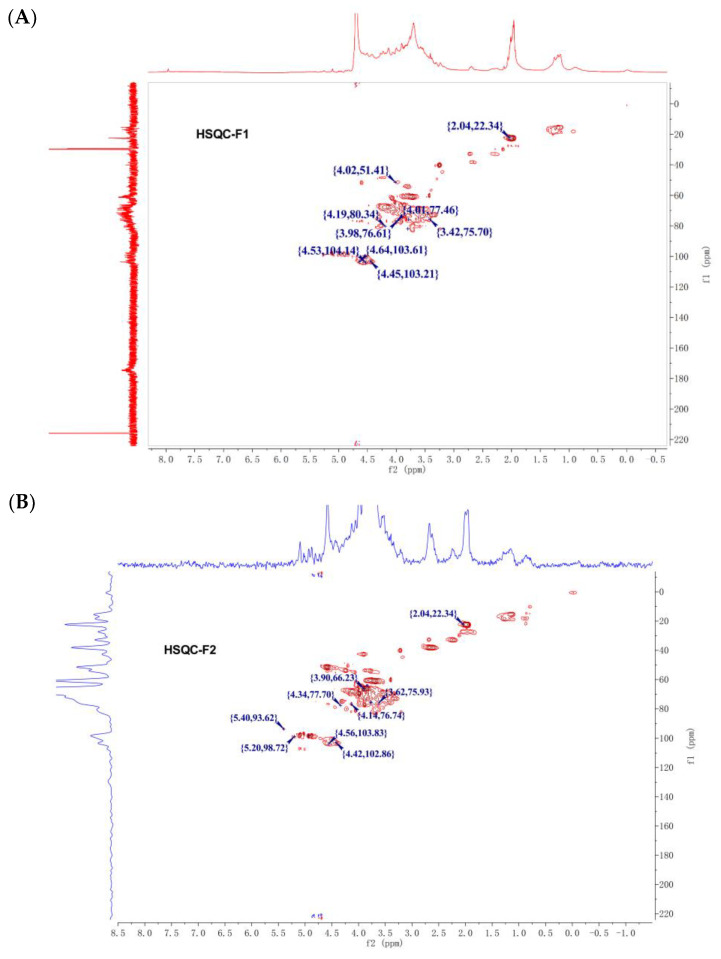
^1^H/^13^C HSQC spectrum of F1 (**A**), F2 (**B**).

The structures of F1 and F2 were further elucidated using ^1^H and ^13^C NMR and combined with results from disaccharide composition and methylation analysis. The chemical shifts of individual residues were assigned according to HSQC experiments (Figure 4). F1 is composed of two types of residues. The chemical shifts of multiple protons on the sugar ring are 3–5 ppm, and 2.04 ppm indicates the presence of methyl in GalNAc [23]. The low field signal of 174.35 ppm and 173.8 ppm indicates the presence of carboxyl groups of acetamido and hexuronic acid, and the high field signal of 22.45 ppm may be acetamide methyl carbon. Detailed attribution is shown in Table 3, and it was speculated that the main repeating unit was →4GlcA→3GalNAc→, which was similar to the chondroitin sulfate chain. F2 contains four types of constituent residues, and the signals at 5.40, 5.21, 4.56, 2.04 ppm chemical shifts corresponding to GlcN, IdoA, GlcA, GlcNAc, respectively [17,24]. Detailed attribution is shown in Table 3, and it was speculated that the main repeating unit was →GlcA (or IdoA) →4GlcN (or GlcNAc) →, which was similar to the hybrid heparin/heparan sulfate. Sulfate groups exist in the structure of F1 and F2, but their linkages have yet to be determined.

### 3.5. Anticoagulant Activity of Heparinoids from Shrimp Head

**Figure 5:**
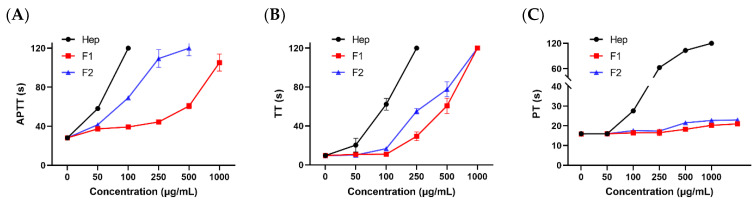
Analysis of APTT (**A**), TT (**B**) and PT (**C**) of F1 and F2.

It is critical to evaluate the anticoagulant effect of heparinoid in vitro using the APTT, PT, and TT assays [25]. As shown in Figure 5, there was a dose-dependent relationship among the anticoagulant activity of F1 and F2, which could effectively prolong APTT and TT in the experimental concentration range, indicating that F1 and F2 could exert anticoagulant activity through endogenous and common pathways. There were weak effects of F1 and F2 on PT, indicating F1 and F2 had no significant influence on extrinsic coagulation system. In addition, compared with heparin, a higher concentration was required to achieve the same anticoagulant effect, indicating that the anticoagulant activity of F1 and F2 was weaker than that of heparin.

The anticoagulant potentials of two heparinoids derived from shrimp heads were investigated. As shown in Table 4, the anti-FXa activity, anti-FIIa activity and anti-FXa/anti-FIIa ratio of F1 were 134.26 IU/mg, 139.07 IU/mg and 0.965, respectively, while the anti-FXa activity, anti-FIIa activity and anti-FXa/anti-FIIa ratio of F2 were 227.32 IU/mg, 194.39 IU/mg and 1.17, respectively. As the concentration of heparinoid F1 and F2 increased, more AT-III was activated, which resulted in more Xa, IIa binding to AT-III and eventually inactivation. The above results indicated that the anticoagulant effect of heparinoid F1 and F2 is partly consistent with the anticoagulant mechanism of heparin. At the same time, the acceleration of F1 and F2 on the inactivation of thrombin and coagulation factors through AT-III is also a potential mechanism. Gomes et al. [7] examined the anticoagulant activity of heparin derived from porcine intestinal mucosa and bovine intestinal mucosa and found that there were significant differences in anticoagulant activity among them. Bovine heparin exhibited anti-FXa and anti-FIIa activities of 92.2 IU/mg and 88.1 IU/mg, with a ratio of 1.04, while the anti-FXa and anti-FIIa activities of porcine heparin were 204.6 IU/mg and 216.2 IU/mg, with a ratio of 0.98. Bovine heparin requires twice the dose of porcine heparin to achieve the same anticoagulant effect, implying that heparin from different sources significantly differed in activity.

### 3.6. Hemorrhagic Effects

**Figure 6:**
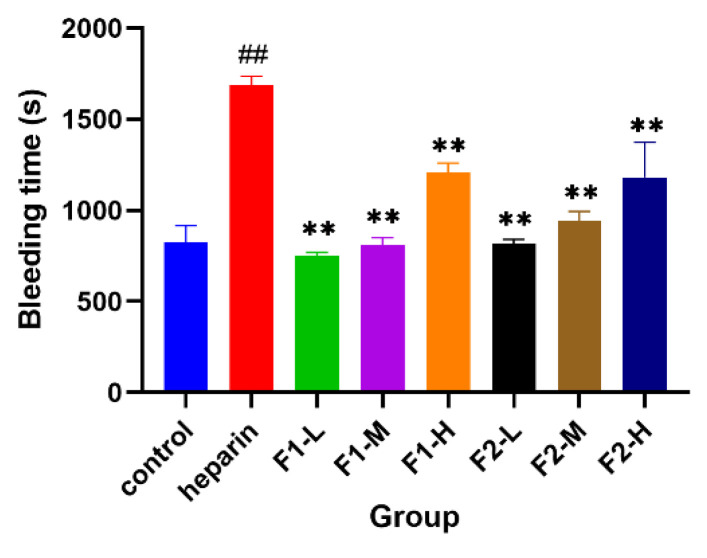
Bleeding effect of F1 and F2. ## *p* < 0.01 vs. control group; ** *p* < 0.01 vs. heparin group (*n* = 6).

The ideal antithrombotic drug should prevent thrombosis without raising bleeding risks [15], so evaluating the bleeding effect is a very important issue. Bleeding time is the time required to begin bleeding to natural coagulation after capillary damage. The time required to hemostasis after mouse tail amputation is often used to evaluate bleeding side effects of samples. As shown in Figure 6, compared with the saline group the high-dose group of F1, F2, and heparin group, as well as the medium-dose group of F2 could significantly prolong the tail bleeding time of mice. Compared with the heparin group, the bleeding time of F1 and F2 in mice was significantly lower than that of the heparin group. It showed that F1 and F2 have better safety and less bleeding side effects.

### 3.7. Fibrinolytic Activity In Vitro

**Figure 7:**
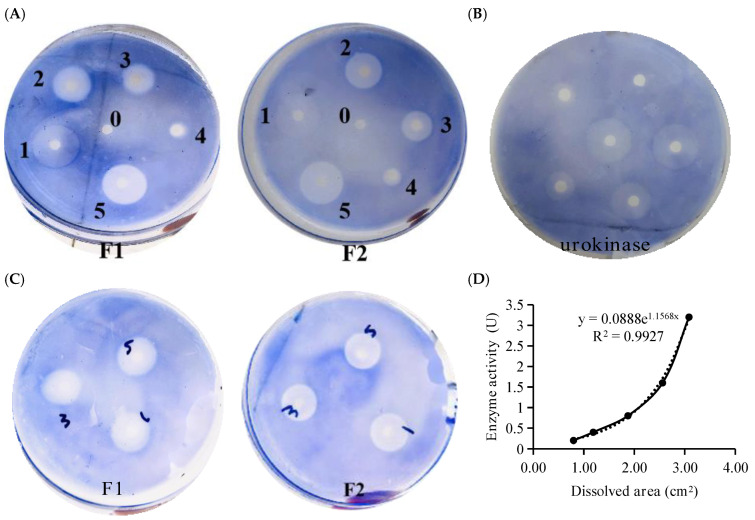
Fibrinolysis evaluation of F1 and F2 in vitro, 0-PBS, 1–200 U/mL Urokinase, 2–10 mg/mL, 3–5 mg/mL, 4–1 mg/mL, 5-Hep 10 mg/mL (**A**). Dissolution ring area produced by different concentrations of urokinase (**B**). Results of 10 mg/mL dissolution circle of F1, F2 (**C**). Urokinase standard curve (**D**).

Heparinoids F1 and F2 have good fibrinolytic activity in vitro (Figure 7A). The size of the dissolution ring increased by the concentrations of F1 and F2, indicating a concentration dependence. The area of the dissolved ring produced by different urokinase concentrations was used to draw the exponential growth curve (Figure 7B). The dissolved ring produced by F1, F2 at 10 mg/mL is shown in Figure 7C, and the average areas are 2.34 cm^2^ and 2.18 cm^2^. The fibrinolytic activity of F1, F2, and heparin is 6.67 ± 0.17 U/mg, 5.52 ± 0.54 U/mg and 9.33 ± 0.82 U/mg, respectively (Figure 7D), indicating a higher fibrinolytic activity of heparin than F1 and F2. Overall, F1 has a better in vitro ability to dissolve fibrinogen than F2.

### 3.8. Effect on Thrombosis and Fibrinolytic Activity In Vivo

Table 5 shows that there was no difference in body weight between the mice among each group after one week of adaptive feeding. Furthermore, the body weight of the mice in each group increased significantly after normal saline or heparinoid injections during the administration period (*p* < 0.05), and there was no significant difference in body weight between the groups indicating that heparinoid had no effect on normal mouse growth.

**Figure 8:**
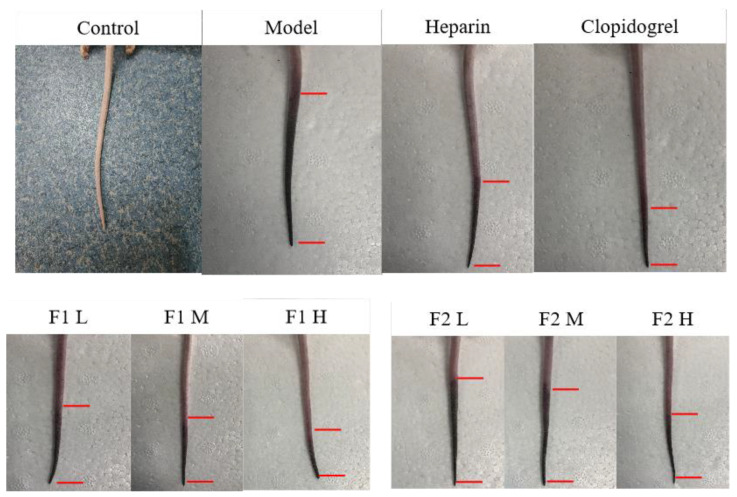
Effects of heparinoid F1 and F2 on carrageenan-induced thrombosis in mice.

**Figure 9:**
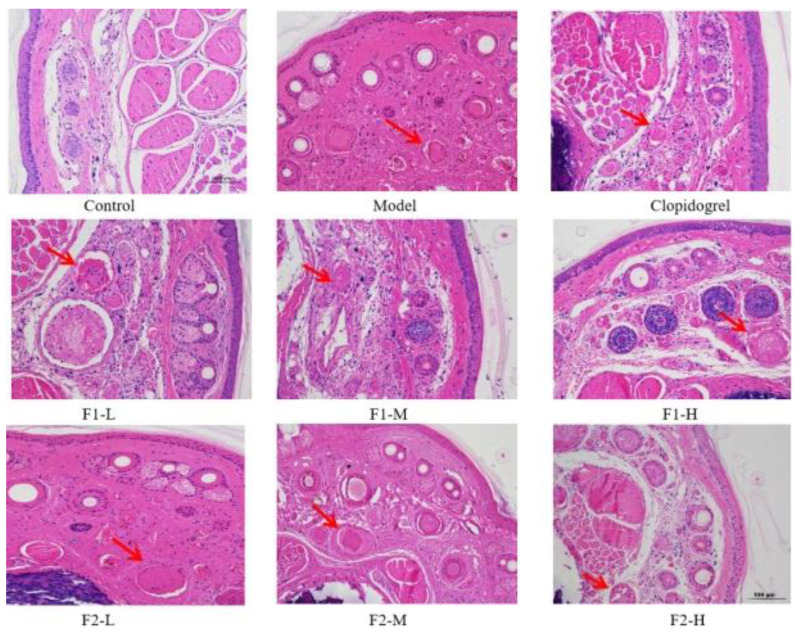
Effects of heparinoid on the histomorphology of tail-vein thrombus.

**Figure 10:**
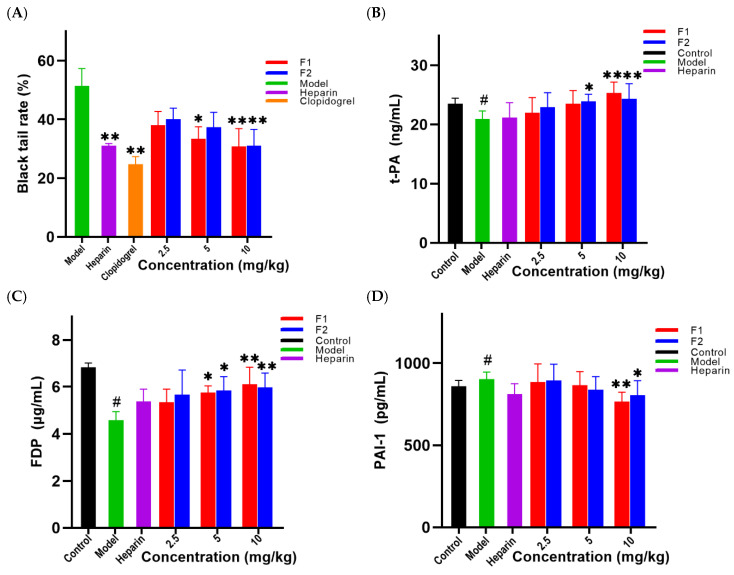
At the end of 48 h (**A**) in different treatment groups, the thrombosis rate was calculated (the ratio of the tail length with thrombus to the whole tail length), the content of t-PA, PAI-1 and FDP (**B**–**D**) in the plasma of mice in different treatment groups (# *p* < 0.05 vs. control group; * *p* < 0.05, ** *p* < 0.01 vs. Model Group).

Anti-thrombotic activity was commonly evaluated using thrombosis in mice tail induced by carrageenan [18]. Mice tail thrombosis occurs 24 h after carrageenan injection with the appearance of dark red. To eliminate the influence of the experimental animals’ tail lengths, the percentage of the black tail in the total tail of each mouse was used as the evaluation index. According to Figure 8, there was no thrombosis in the tails of the mice in the blank group, but varying degrees of thrombosis were formed in the tails of the mice in the model group, heparin group, clopidogrel group, and heparinoid F1 and F2 groups (low, medium, and high dose). The black tail ratio results after 24 and 48 h of modeling are shown in Figure 10A; when compared to the model group, significant differences were found in the middle and high dose groups of heparinoid F1 and F2 (*p* < 0.05). The length of the black tail in mice was significantly shortened by dose in a dose-dependent manner. The H&E staining results confirmed the black tail ratio (Figure 9); there was no thrombus in the control group and the vascular distribution of the tail was clearly visible. However, the tail-vein was blocked to varying degrees in each treatment group (injected with carrageenan). In the mouse tail-vein treatment with high doses of F1 and F2, less vascular blockage was observed compared to the model group. These findings demonstrated that heparinoid F1 and F2 could effectively slow and prevent tail thrombosis in mice.

Figure 10 depicts the results of determining some fibrinolytic system indexes in mice using heparinoids F1 and F2. Heparinoid F1 and F2 can significantly increase t-PA and FDP levels in mouse plasma with a dose-effect relationship. The content of t-PA and FDP in the plasma of the model group decreased significantly when compared to the blank group, indicating that the body’s fibrinolytic function was compromised. FDP content in the model group was 33.18% lower than in the blank group and t-PA content decreased by 10.78%. The development of black tail thrombosis in mice disrupted normal fibrinolytic function. Compared with the model group, the content of t-PA in the plasma of the high dose group of F1 and F2 was significantly higher than that of the low dose group and the model group. The high dose group of F1 could increase the content of t-PA by 21.01% (*p* < 0.01), and the medium and high dose groups of F2 could increase the content of t-PA by 13.99% and 16.28%, respectively (*p* < 0.05), indicating that the injection of a high dose of F1 and F2 could significantly increase the content of t-PA in the plasma of mice.

FDP, an indicator that reflects the hyper fibrinolysis in vivo, was significantly higher in the middle and high dose groups of F1 and F2 than that of model and low dose groups. The middle and high dose groups of F1 could increase the content of FDP by 25.60% and 33.69%, respectively. FDP content could be increased by 25.60% and 33.69% in the middle and high dose groups of F1. FDP content in middle and high dose groups of F2 increased by 27.29% and 30.85%, respectively. The mice initiated their own fibrinolytic system and the amount of FDP generation increased. A low dose of F1 and F2 shrimp head heparinoid had no effect on the fibrinolytic system in mice. F1 and F2 shrimp head heparin at medium and high doses can improve the ability of the fibrinolytic system in mice with a dose-effect relationship.

PAI-1 is the primary t-PA inhibitor, and it can prevent the dissolution of tissue and plasma fibrin [26]. PAI-1 release can be effectively inhibited by heparinoids F1 and F2. The content of PAI-1 released when heparinoids F1 and F2 were administered at a dose of 10 mg/kg differed significantly from that of the model group. F1 can reduce PAI-1 content by 15.17%, while F2 can reduce it by 10.96% and the improvement effect of F1 and F2 low-dose groups is unclear. The results showed that heparinoids F1 and F2 could prevent thrombus formation by significantly increasing the content of plasma t-PA in mice and the content of FDP, inhibiting the release of PAI-1 and promoting fibrinolysis function.

An imbalance between coagulation and anticoagulation, as well as abnormal coagulation-fibrinolysis, can result in thrombus formation [27]. The effects of two kinds of heparinoid on thrombosis were investigated from the perspective of anticoagulation and fibrinolysis in this study. Although the anticoagulant activity of marine-derived heparinoid was lower than that of mammalian-derived heparin, it has less toxicity and side effects (such as bleeding), is relatively safe, and has clinical application value [28] as confirmed by our study. This was supported by an earlier observation by Brito et al. [29], who demonstrated that shrimp heparin contains more glucuronic acid and sulfated disaccharide units than pig intestine heparin, and its anticoagulant activity is significantly lower. However, the anticoagulation mechanism of heparinoid in this study is slightly different, as chondroitin sulfate from shrimp head had no effect on inhibition of factor Xa [30], which may be due to the inability to stabilize AT. On the contrary, the anticoagulant mechanism of two heparinoids in this study may relate to their combination with more AT-III, as well as inhibition of coagulation factors Xa and IIa and prevention of blood clot formation. Furthermore, this is the first report of two heparinoids derived from shrimp heads activating fibrinolytic activity by increasing t-PA and FDP content while decreasing PAI-1 levels. In view of the structural complexity of F1 and F2, the relationship between their activity and structure needs to be further studied.

## 4. Conclusions

Heparins and heparinoids as bioactive macromolecules are both classical and prospective. These are extracted from many animal sources including mammals and marine organisms. Heparinoids prepared from different animal sources vary in properties, structure and activity. In this study, we have indicated the thrombosis preventive potential of heparinoid compounds isolated from shrimp heads through a series of activity evaluations in vitro and in vivo (anticoagulant, fiber system balance, thrombosis, and effects on thrombosis).

In the present study two heparinoids, F1 and F2, were extracted from shrimp head, and their molecular weights were 7.8 and 19.8 kDa, respectively. F1 is thought to have a structure similar to chondroitin sulfate, while F2 has a structure similar to hybrid heparin/heparan sulfate. F2 has a higher molecular weight and exhibited stronger anticoagulant effect, but F1 showed stronger fibrinolytic capability. Compared to mammalian heparin, both F1 and F2 demonstrated a high ability to prevent thrombosis, primarily through anticoagulation and activation of the body’s own fibrinolytic system and while posing a lower risk of bleeding. Overall, this work strongly suggests bioactive ingredients derived from shrimp heads be investigated in the healthcare industry, particularly heparinoid which can offer a promising strategy for utilizing shrimp processing waste.

## Figures and Tables

**Table 1:** Analysis of disaccharides composition of F1 and F2.

	ΔUA2S-GlcNS, 6S	ΔUA2S-GlcNA_C_, 6S	ΔUA-GlcNS, 6S	ΔUA-GlcNA_C_, 6S	ΔUA2S-GlcNS	ΔUA2S-GlcNA_C_	ΔUA-GlcNS	ΔUA-GlcNA_C_	IIA-IIS_glu_
Hep	63.79%	2.12%	9.72%	2.87%	6.78%	0.4%	2.87%	2.95%	2.26%
F1	13.18%	-	26.56%	1.68%	16.64%	2.29%	7.97%	10.93%	2.48%
F2	12.36%	-	28.97%	3.49%	9.93%	11.17%	6.39%	7.97%	1.71%

- Not detected.

**Table 2:** Linkage types of F1 and F2.

RT	Methylated Sugar	Molar Ratio	Type of Linkage	Mass Fragments (*m*/*z*)
16.458	2,3,5-Me3-Araf	0.018	Araf-(1→	43, 71, 87, 101, 117, 129, 145, 161
19.064	2,4-Me2-Araf	0.035	→3)-Araf-(1→	43, 85, 99, 101, 117, 127, 161, 159
22.147	2,3-Me2-Araf	0.039	→5)-Araf-(1→	43, 71, 87, 99, 101, 117, 129, 161, 189
22.667	2,3,4,6-Me4-Glcp	0.208	Glcp-(1→	43, 71, 87, 101, 117, 129, 145, 161, 205
24.057	2,3,4,6-Me4-Galp	0.031	Galp-(1→	43, 71, 87, 101, 117, 129, 145, 161, 205
29.813	2,3,6-Me3-Glcp	0.431	→4)-Glcp-(1→	43, 87, 99, 101, 113, 117, 129, 131, 161, 173, 233
30.342	2,4,6-Me3-Galp	0.043	→3)-Galp-(1→	43, 87, 99, 101, 117, 129, 161, 173, 233
33.712	2,6-Me2-Glcp	0.054	→3,4)-Glcp-(1→	43, 87, 97, 117, 159, 185
39.077	2,4-Me2-Galp	0.141	→3,6)-Galp-(1→	43, 87, 117, 129, 159, 189, 233
RT	Methylated sugar	Molar ratio	Type of linkage	Mass fragments (*m*/*z*)
32.968	2,3,4,6-Me4-Glcp	0.698	→4)-β-D-GlcpA-(1→	43, 87, 99, 101, 113, 117, 129, 131, 161, 173, 233
46.741	2,3,6-Me3-Glcp	0.302	→3)-β-D-GlcpNAc-(1→	43, 75, 100, 117, 129, 158, 171

**Table 3:** ^1^H and ^13^C NMR chemical shifts of F1 and F2.

Residue	1	2	3	4	5	6	NAc
GlcA	103.31/4.43	69.13/3.59	71.29/3.56	80.11/3.70	72.81	173.8	-
GalNAc	103.59/4.63	61.95/4.08	77.69/4.02	70.56/4.14	73.90/3.84	60.53/3.78	174.35; 22.35/2.04
Residue	1	2	3	4	5	6	NAc
GlcA	103.63/4.56	72.31/3.31	75.75/3.76	69.13/3.68	75.71/3.74	-	-
IdoA	99.52/5.21	77.70/4.34	72.15/3.70	76.74/4.14	74.73/4.93	174.54	-
GlcN	93.62/5.40	75.55/3.78	68.97/3.78	75.93/3.62	74.03/3.73	66.23/3.90	22.34/2.04

**Table 4:** Anti-Xa factor and IIa factor analysis of F1 and F2.

Group	FXa/IU × mg^−1^	FIIa/IU × mg^−1^	Xa/IIa
F1	134.26	139.07	0.965
F2	227.32	194.39	1.17

**Table 5:** The change in mice body weight (*n* = 6).

Group	Begin Body Weight/g	Weight before Model/g
Control	24.94 ± 1.46	33.70 ± 1.90 *
Model	24.68 ± 1.01	35.42 ± 3.38 *
Heparin	26.00 ± 1.74	34.48 ± 2.30 *
Clopidogrel	23.76 ± 0.67	34.40 ± 1.33 *
F1 L	24.66 ± 0.75	35.16 ± 2.08 *
F1 M	24.34 ± 0.86	34.60 ± 1.32 *
F1 H	24.88 ± 1.01	35.86 ± 0.81 *
F2 L	24.86 ± 0.47	36.20 ± 1.49 *
F2 M	25.22 ± 1.32	35.76 ± 1.40 *
F2 H	24.38 ± 1.01	35.48 ± 2.22 *

* *p* < 0.05.

## Data Availability

The data presented in this study are available on request from the corresponding author.
